# Mechanistic characterization of the DEAD-box RNA helicase Ded1 from yeast as revealed by a novel technique using single-molecule magnetic tweezers

**DOI:** 10.1093/nar/gkz057

**Published:** 2019-02-14

**Authors:** Saurabh Raj, Debjani Bagchi, Jessica Valle Orero, Josette Banroques, N Kyle Tanner, Vincent Croquette

**Affiliations:** 1 Laboratoire de Physique de l’Ecole normale supérieure, ENS, Université PSL, CNRS, Sorbonne Université, Université Paris-Diderot, Sorbonne Paris Cité, Paris, France; 2 IBENS, Département de biologie, École normale supérieure, CNRS, INSERM, PSL Research University, 75005 Paris, France; 3 Laboratoire d’Expression Génétique Microbienne, CNRS UMR8261/Université Paris 7-Diderot, Sorbonne Paris Cité Universités, 13 rue Pierre et Marie Curie, Paris, France; 4 Institut de Biologie Physico-Chimique, PSL Research University, 75005 Paris, France; 5 ESPCI Paris, PSL University, 10 rue Vauquelin, 75005 Paris, France

## Abstract

DEAD-box helicases are involved in all steps of RNA metabolism. They are ATP-dependent RNA binding proteins and RNA-dependent ATPases. They can displace short duplexes, but they lack processivity. Their mechanism and functioning are not clearly understood; classical or bulk biochemical assays are not sufficient to answer these questions. Single-molecule techniques provide useful tools, but they are limited in cases where the proteins are nonprocessive and give weak signals. We present here a new, magnetic-tweezers-based, single-molecule assay that is simple and that can sensitively measure the displacement time of a small, hybridized, RNA oligonucleotide. Tens of molecules can be analyzed at the same time. Comparing the displacement times with and without a helicase gives insights into the enzymatic activity of the protein. We used this assay to study yeast Ded1, which is orthologous to human DDX3. Although Ded1 acts on a variety of substrates, we find that Ded1 requires an RNA substrate for its ATP-dependent unwinding activity and that ATP hydrolysis is needed to see this activity. Further, we find that only intramolecular single-stranded RNA extensions enhance this activity. We propose a model where ATP-bound Ded1 stabilizes partially unwound duplexes and where multiple binding events may be needed to see displacement.

## INTRODUCTION

RNA helicases are ubiquitous proteins that are found in all three kingdoms of life and that are associated with all processes involving RNA, from transcription to decay (1–3). Like their DNA helicase counterparts, they are characterized by highly conserved core structures with structural homology to the recombinant protein A (RecA) and that contain highly conserved nucleotide triphosphate binding sites, called the Walker A and B motifs. Most RNA helicases are classified into superfamilies (SFs) 1 and 2, which contain catalytic cores consisting of two, linked, RecA-like domains. Despite their commonly shared cores, these proteins have highly diversified specificities and enzymatic activities. They have been shown to unwind RNA–RNA and RNA–DNA duplexes, displace RNA-bound proteins, remodel ribonucleoprotein complexes and act as RNA chaperones to insure the correct formation of RNA secondary and tertiary structures. Some translocate on RNAs in the 5′ to 3′ direction, others in the 3′ to 5′ direction and some have little or no translocation activity. Nevertheless, on the whole they all can be considered to be NTP-dependent (or NDP-dependent) RNA binding proteins and RNA-dependent NTPases. In general, helicases (both RNA and DNA) show a broad range of helicase activities that can be easily distinguished by single-molecule techniques (4). Some are highly processive and active helicases: these proteins are able to separate duplexes at a constant rate that is independent of the stability of the base pairs. On the opposite extreme, some are passive and opportunistic: they seem to take advantage of base fraying to progress along the polynucleotide chain. Others pause at G/C-rich regions and accelerate in regions that are less stable, and consequently they represent an intermediate activity.

The largest family of RNA helicases is the DEAD-box proteins found in SF2 (5,6). These proteins typically contain a Walker B motif (motif II) sequence that is D-E-A-D in single amino-acid nomenclature, and they use ATP as a cofactor. These proteins have weak, nonprocessive, helicase activity that is highly sensitive to the stability of the duplex. They are thought to unwind duplexes by localized strand disruption at the site of binding (7). There are now a number of solved crystal structures of different DEAD-box proteins bound to the nucleotide and RNA ligands, and they all show the same underlining interactions (6). In the presence of bound RNA and adenosine 5′-(β,γ-imido)triphosphate (AMP-PNP), which is a nonhydrolyzable analog of ATP, the two RecA-like domains form a ‘closed’ conformation with high affinity for RNA. The interactions with RecA-like domain 2 are consistent with RNA (single-stranded or double-stranded) in the form of an A-form helix, although only a single strand of the RNA is actually bound. In contrast, the interactions with RecA-like domain 1 show steric hindrance that would disrupt a helix and unstack the terminal bases. This is considered the primary mechanism by which DEAD-box proteins unwind duplexes. In the absence of ATP or in the presence of ADP, the RecA-like domains are thought to assume an ‘open’ conformation with low affinity for the ligands (6,8).

Unfortunately, the lack of processivity of DEAD-box proteins limits the sensitivity of single-molecule analyses because the region unwound is relatively small, which results in a weak signal. To overcome this limitation of existing assays, we have developed a new assay using single-molecule magnetic tweezers that amplifies the effects of the displacement of a short duplex and reveals even weak helicase activity. We tested this system with the yeast Ded1 protein, which belongs to the DDX3 subfamily of DEAD-box proteins (9–13). These experiments differ from previously reported single-molecule experiments of Ded1 that used atomic force microscopy because duplex unwinding was independent of the applied force and because we could easily modify the experimental parameters (14). By studying Ded1 activity on various types of substrates at the single molecule level, we show that Ded1 has high affinity for single-stranded RNA, is nonprocessive and that it unwinds duplexes by local melting of a few base-pairs. On the basis of our results, we propose a model for the molecular mechanism of the unwinding activity that is consistent with our results and previously published data.

## MATERIALS AND METHODS

### Experimental setup

A PicoTwist magnetic tweezers instrument was used to manipulate individual DNA hairpins tethered between magnetic beads and a coverslip. The magnetic bead was held by a force due to a vertical magnetic field gradient generated by a pair of permanent magnets. Precisely controlling the distance of the magnets from the sample surface varied the force on the samples. The movement of these magnets was achieved with the help of PI DC-motor, which allowed us to exert a force with sub-picoNewton accuracy. The force was calculated from the Brownian fluctuations of the tethered bead (15).

The DNA substrate used in the single-molecule studies consisted of a 1239 bp hairpin with a four-nucleotide-loop, a 76-nucleotide-long, 5′-biotinylated, ssDNA tail and a 146 bp 3′-digoxigenin-labeled dsDNA tail (see Supplementary Figure S1). The 1.2 kbp hairpin was synthesized and attached as previously described (16). In brief, we attached this hairpin at one end to a streptavidin-coated magnetic bead through a biotin-streptavidin bond and at the other end to a coverslip through a digoxigenin-anti-digoxigenin bond.

A CMOS camera at the image plane of the objective was used to track the position of the tethered magnetic bead in three dimensions with nanometer resolution at 30 Hz. Thus, tracking the z-axis fluctuations of the tethered bead enabled us to monitor the change in extension of the tethered DNA hairpin in real time with an accuracy of about 5 nm. The extension of the DNA changed in accordance with the applied force, by the presence of hybridized oligonucleotides and by added Ded1 protein. Tens of beads were tracked simultaneously in real time in order to obtain statistically significant measurements of single molecule events. More details about the experimental setup are mentioned elsewhere (17). The determination of the ssDNA elasticity is described in Supplemental Figure S2.

The working buffer used in all the helicase assays for Ded1 was composed of 20 mM Tris–HCl, pH 7.5, 50 mM potassium acetate, 3 mM magnesium acetate, 2 mM dithiothreitol and 0.2 mg/ml bovine serum albumin. All the single-molecule experiments were conducted at a temperature of 30°C.

### Hairpin refolding blockage and ssDNA binding assay

A DNA hairpin was unfolded by pulling a magnetic bead on one end of the strand while the other end was tethered to a glass surface. The hairpin became unfolded and fully extended at a force larger than 15 pN, and it instantaneously refolded, when the force was reduced, through initial base pairing at the region corresponding to the apex of the hairpin. The net extension thereby became zero. However, an oligonucleotide hybridized to the apical region, during the phase when the DNA was extended, blocked the reformation of the hairpin when the force was reduced. As a consequence, the extension of the hairpin was transiently blocked for a finite value before the hairpin fully refolded. Adjusting the force during the refolding provided a way to detect weak binding. The blocking event strongly depended on whether the oligonucleotide bound in the loop apex or in the stem region of the DNA molecule. In the first case, the blocking was stronger because nucleation of the hairpin was kinetically blocked. This represented several k_B_T units (Boltzmann constant multiplied by the temperature in K; 1 *k*_B_*T* = 4.18 pN·nm at 30°C). In the latter case, oligonucleotides were more easily displaced because the refolding hairpin exerted a displacement force.

In our assay, we programmed the magnetic-tweezers device to periodically open and close the hairpin with a short opening time and a longer refolding time; we then studied the refolding signals to determine how many blockages were observed versus the number of cycles. This assay detected single binding events. We could alter the conditions by varying the force and by adding various factors, such as a helicase and ATP, that altered the observed off rates, *T*_off_.

### Data collection and analysis

The image of the bead displayed diffraction rings that were used to estimate its three dimensional position as explained elsewhere (15). From the fluctuating positions of the bead both the mean elongation of the molecule and the force applied to it could be deduced (18). The z-axis fluctuations were acquired at 30 Hz. We drew the cumulative histogram of the number of events that lasted longer than *T*_hold_ in order to measure the mean displacement rate (|*T*_off_|) of the oligonucleotide when the duplex was very stable and most of the displacement times (*T*_off_) exceeded the holding force time (*T*_hold_); this function was an exponential with a characteristic time |*T*_off_|, and it represented a classical histogram of the number of events in a bin that presented an exponential behavior except for the last bin, which corresponded to all the events lasting more than *T*_hold_.

### Oligonucleotides

The following oligonucleotides were used, where base-paired regions to the DNA are underlined and DNA nucleotides indicated with a ‘d’: 11bp (5′ AGA UGC CAA AA 3′); 5′ss-11 bp (5′ AAA AAA AAA AAA GAU GCC AAA A 3′); 11 bp-3′ss (5′ AGA UGC CAA AAA AAA AAA AAA A 3′); 15 bp (5′ C GUC AGA UGC CAA AA 3′); 5′ss-15 bp (5′ AAA AAAAAA AAC GUC AGA UGC CAA AA 3′); 15bp-3′ss (5′ CGU CAG AUG CCA AAA AAA AAA AAA AA 3′); DNA-11 bp (5′ dAdGdA dTdGdC dCdAdA dAdA 3′); 11 bp-2R-9D (5′ AGdA dTdGdC dCdAdA dAdA 3′); 11 bp-3R-8D (5′ AGA dTdGdC dCdAdA dAdA 3′); 11 bp-4R-7D (5′ AGA UdGdC dCdAdA dAdA 3′); 11 bp-8D-3R (5′ dAdGdA dTdGdC dCdAA AA 3′); 11 bp-7D-4R (5′ dAdGdA dTdGdC dCAA AA 3′); 11 bp-4D-2R-5D (5′ dAdGdA dTGC dCdAdA dAdA 3′); 11 bp-4D-4R-3D (5′ dAdGdA dTGC CAdA dAdA 3′); RNA01 (5′ UCA UAC UUU UCU UUU CUU UUC CAU C 3′); RNA02 (5′ GAU GGA AAA GAA AAG AAA AGU AUG A 3′); 5′ss-30 bp (5′ AAA AAA AAA AAA AAA GAU AAG CCU ACU ACA GUA GAU UUU GAC GGG 3′). The two mixed RNA–DNA oligomers, where the RNA–RNA interactions are underlined, used to form the cruciform consisted off: 5′ GAG CGU CAG CdCdC dAdCdC dAdTdT dCdAdC dAdTdG dCdTdT dAdGdG dAdGdC dGdG 3′ and 5′ dCdGdG dTdTdA dGdTdT dTdCdC dGdCdT dCdCdT dAdAdG dCdAdT dGdTdG dAdAdT dGdGdT dGdGG CUG ACG CUC 3′.

### Recombinant protein expression and purification

Ded1-His6 was expressed in pET22b (Novagen) and purified as previously described (19,20). The purity was verified by separating the material by electrophoresis on a SDS, Laemmli, polyacrylamide gel. The protein concentrations were determined by the Bio-Rad Protein Assay using bovine serum albumin as a standard. The activities were verified by measuring the RNA-dependent ATPase activities using whole yeast RNA as previously described (21). Ded1 was aliquoted in small portions and stored at –80°C in 50% glycerol until needed. To insure reproducibility, aliquots were thawed once and discarded after each experiment.

## RESULTS

### Single-molecule magnetic tweezers experimental system

The experimental system was based on manipulating multiple single-stranded DNAs (ssDNA) that were attached to a glass slide on one end and magnetic beads on the other (4). The DNA contained extensive regions of self-complementarity that resulted in an extended hairpin. However, under sufficient force the DNA could be extended to form a single-stranded chain. This unwound DNA was subsequently stabilized by hybridizing an oligonucleotide of interest at the region corresponding to the apex of the hairpin, which blocked the refolding of the hairpin when the force was reduced. The eventual displacement of this oligonucleotide was followed over time by the spontaneous reformation of the DNA hairpin that was readily seen by the large differences in extension between the two forms. We were able to monitor a field of multiple molecules at the same time to obtain statistically significant measurements of the enzymatic activities under various conditions.

**Figure 1.**
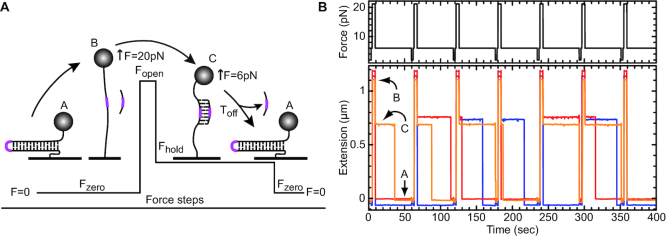
Principle of the assay done with magnetic tweezers. (**A**) The assay involved three phases and three possible molecule states (A, B and C; described in detail in the text). In brief, the force value imposes the molecular state. At phase one, the force was close to 0 pN (*F*_zero_), the hairpin was completely folded (state A), and its extension was defined as zero. At phase two, the force was increased (*F*_open_) to about ∼20 pN for a few seconds, and the hairpin was completely unwound and the DNA fully extended (state B). An oligonucleotide (RNA or DNA) complementary to the apical region of the DNA hairpin (shown in magenta) was able to hybridize to the DNA after a few seconds at F_open_. At phase three, the force was reduced to 6 pN (F_hold_); however, the bound oligonucleotide prevented the DNA hairpin from reforming, and the DNA remained in a transiently extended (state C). During this phase, the oligonucleotide could dissociate from the DNA and return the molecule to state A, where the duration of state C was defined as *T*_off_. Finally, the force was reduced to zero (*F*_zero_), which caused any remaining molecules in state C to refold, and a new cycle was started. These phases were repeated numerous times with the same DNA molecule. (**B**) An example of the experimental assay. The top panel shows the force modulation acting on the hairpin while the lower panel shows the real time extension of three individual hairpins (red, blue, orange) with respect to the force applied. The red and blue traces were displaced slightly up and down, respectively, to facilitate viewing. The force modulation cycles were repeated ∼100–150 times with about 30 to 50 beads to obtain statistically significant values of the mean *T*_off_, |*T*_off_|.

The assay consisted of applying periodic force cycles over three major phases that imposed three molecular states (Figure 1A). In phase I, the force was set at ∼20 pN (*F*_open_) for a few seconds to convert the DNA hairpin into an extended single strand (Figure 1A, state A to B). An oligonucleotide present in the chamber then hybridized at the apical position of the hairpin. In phase II (Figure 1A, state B to C), the force was decreased to ∼5–6 pN (F_hold_) for a fixed time (*T*_hold_). At *F*_hold_, the DNA hairpin spontaneously refolded in the absence of the oligonucleotide (state A). In its presence, the DNA remained extended as long as the oligonucleotide remained bound; the molecule extension was slightly reduced due to the elasticity of the ssDNA (state C). We defined the refolded state as zero extension. Thus, a 1.2 kb hairpin extended to ∼1.2 μm when *F* = *F*_open_, reduced to ∼0.75 μm when *F* was set at *F*_hold_ and an oligonucleotide was hybridized, and then to zero when the oligonucleotide was displaced (Figure 1b). The transition from state C to A gave the displacement rates (*T*_off_) of the oligonucleotides at *F*_hold_. Finally, in phase III the force was reduced to ∼0.5–1 pN to eject any remaining oligonucleotide (Figure 1A). This cleaning phase ensured that all the cycles were independent. A typical experiment is shown in Figure 1B for several DNA molecules.

This cyclic assay provided several important advantages. First, the bound oligonucleotide did not need to be labeled. Second, the force in phase II (*F*_hold_) was adjusted so the extension of the ssDNA was equal to that of a B-form DNA–DNA helix or of an A-form RNA–DNA helix, so that there was no mechanical stretching energy contribution involved in the hybridization process (22, see Supplementary Figure S2). Third, the transition from elongated DNA to closed hairpin amplified the signal and revealed the displacement of even short oligonucleotides. Another advantage of this assay was that the ssDNA was not a substrate for the protein; DEAD-box proteins have only weak, ATP-independent affinity for ssDNA (23). Moreover, the contacts with the RNA substrate in all the solved crystal structures of DEAD-box proteins involves only a single strand of the RNA; the complementary strand can be either RNA or DNA (6). Indeed, it has been shown that both RNA–RNA and RNA–DNA duplexes are good substrates for DEAD-box proteins (e.g. 19,24).

The observed rate of displacement of the oligonucleotide was directly related to the dissociation constant (*k*_off_) because the fast irreversible refolding of the DNA hairpin prevented rehybridization. Thus, *T*_off_ displayed an exponential distribution that could be fit to the equation *P*(*t*) = a*exp(–*t*/|*T*_off_|), where *t* is time, *a* is a constant and |*T*_off_| is the mean off rate (|*T*_off_| = 1/*k*_off_). This value was highly dependent on the G/C content and on the length of the oligonucleotide. In general, we found empirically that |*T*_off_| was reduced by a multiple of approximately three to four for each base pair eliminated. For an RNA oligonucleotide, |*T*_off_| spanned a fraction of a second for a 7-mer and reached ∼1000 s for a 15-mer. On some occasions, *T*_off_ exceeded the hold time, *T*_hold_, used in phase II. In these cases, fitting the histogram with the last bin summing all events exceeding *T*_hold_ enabled us to determine the |*T*_off_|, as long as *T*_hold_ was not too short (see methods). In contrast, the hybridization time (*T*_on_) was dependent on the oligonucleotide concentration. We typically used nanomolar concentrations of oligonucleotide that yielded high ratios to the tethered DNA and added enzyme.

The |*T*_off_| measured provided a proxy to detect a helicase activity: destabilizing the oligonucleotide reduced |*T*_off_| significantly. Thus, the ratio |*T*_off_| /|*T*_off_ [H]|, where [H] is the helicase concentration, was a measure of the enzymatic activity. However, this ratio was not entirely proportional to this activity because it did not take into account the time it takes for the protein to bind to the substrate, which depended on the concentration-dependent diffusion of the protein and its correct positioning on the substrate. Therefore, the ratio was a reflection of both protein binding and helicase activity, and it would vary according to the duplex stability.

### The DEAD-box helicase Ded1 can efficiently displace RNA oligonucleotides

To test our system we chose the DEAD-box helicase Ded1 from yeast. It has previously been shown to be one of the most active DEAD-box proteins with the capacity to unwind, in an ATP-dependent fashion, a large excess of substrates at nanomolar concentrations (25). Both RNA–RNA and RNA–DNA duplexes are efficiently disrupted as long as any single-stranded regions are RNA (19). Others found that flanking ssDNA also could enhance the activity but only at very large concentrations of Ded1 relative to the substrate (26). These regions provide ‘landing sites’ that greatly enhance the enzymatic activity, but they are found to be equally effective either 5′ or 3′ to the duplex. Consequently, Ded1, like most characterized DEAD-box proteins, does not show any directionality. The unwinding activity is highly sensitive to the duplex stability; for example, it was shown that a 50-fold excess of a 16 bp duplex, with a calculated Gibbs free energy of –19.8 kcal/mol (–82.8 J/mol), is completely displaced in an ATP-dependent manner in under five minutes at 30°C by Ded1, while a duplex with a value of –25.3 kcal/mol (-106 J/mol) is not completely disrupted even after 30 min with an equimolar concentration of protein and substrate (19).

**Figure 2.**
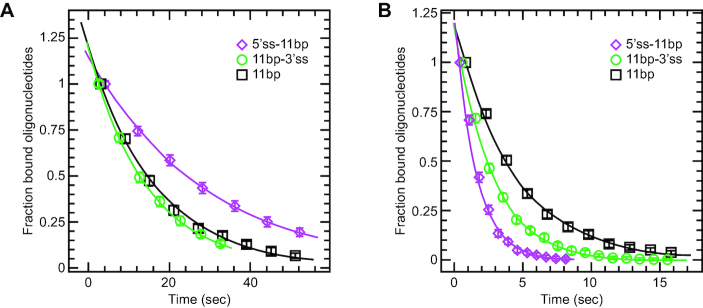
The rates at which oligonucleotides were displaced from the extended DNA at F_hold_ (Figure 1A, state C to A) in the presence or absence of Ded1. The RNA oligonucleotides all formed the same 11 bp duplex at a region corresponding to the apex of the DNA hairpin. The 5′ss-11bp had an 11-nucleotide-long, single-stranded region on the 5′ end. As a consequence, it could form an additional G-A base pair on the duplex end. The 11 bp-3′ss had an 11-nucleotide-long, single-stranded region on the 3′ end, and the 11bp had no single-stranded RNA extensions. The oligonucleotides were used at 200 nM and the *F*_hold_ was maintained at 6 pN. Each datum point consisted of ∼30 hairpins that were subjected to ∼50 opening and closing cycles. This gave us a large pool of values that gave statistically significant measurements. The distributions were fit to the equation, *P*(*t*) = a*exp(–*t*/|*T*_off_|), where *t* is time, a is a constant and |*T*_off_| is the mean off rate. The data points represent the mean and standard deviations of oligonucleotides displaced for each time interval. (**A**) The intrinsic off rates of the oligonucleotides in the absence of protein. (**B**) The off rates in the presence of 10 nM Ded1 and 1 mM ATP.

We tested three RNA oligonucleotides that could form 11 bp RNA–DNA duplexes at the region corresponding to the apex of the DNA hairpin. One oligonucleotide had a 5′ single-stranded extension consisting of (A)_11_ (5′ss-11 bp), the second had the equivalent extension on the 3′ end (11 bp-3′ss), and the third formed a blunt-end duplex (no ssRNA extension; 11 bp). We tested these oligonucleotides in the absence of protein (Figure 2A; Table 1). Both 11 bp and 11 bp-3′ss had similar |*T*_off_| of around 15–17 s, while the |*T*_off_| of the 5′ss11 bp oligonucleotide was nearly twice longer (Figure 2A; Table 1). This was probably because the 5′ extension could form an additional G-A bp at the end of the duplex. Thus, the intrinsic (protein-independent) displacements of the oligonucleotides were consistent with the anticipated stabilities of the duplexes.

**Table 1.** The off rates for hybridized oligonucleotides

Oligo (type^a^)	Oligo length (bp+flanking^b^)	[Ded1] (nM)	|*T*_off_|(-ATP)^c^ (s/event)	|*T*_off_|(+ATP)^d^ (s/event)	Relative^e^ (-ATP)	Relative^e^ (+ATP)
RNA	11 bp	0	16.9 ± 0.3	NA^f^	NA	NA
RNA	11 bp	10	ND^g^	4.30 ± 0.13	ND	3.93
RNA	5′ss-11 bp	0	28.0 ± 0.8	NA	NA	NA
RNA	5′ss-11 bp	10	18.0 ± 0.5	1.30 ± 0.05	1.56	21.5
RNA	5′ss-11 bp	50	9.10 ± 0.27	<0.1	3.08	> 28
RNA	5′ss-11 bp	100	2.70 ± 0.07	<0.1	10.4	> 28
RNA	11 bp-3′ss	0	14.7 ± 0.44	NA	NA	NA
RNA	11 bp-3′ss	10	ND	2.50 ± 0.08	ND	5.88
RNA	15 bp	0	820. ± 80.	NA	NA	NA
RNA	15 bp	20	ND	3.30 ± 0.07	ND	248
RNA	5′ss-15 bp	0	989. ± 168	NA	NA	NA
RNA	5′ss-15 bp	10	976. ± 65	13.6 ± 0.6	1.01	72.8
RNA	5′ss-15 bp	20	ND	3.00 ± 0.05	ND	330
RNA	15 bp-3′ss	0	1030. ± 80	NA	NA	NA
RNA	15 bp-3′ss	20	ND	2.70 ± 0.05	ND	381
DNA	11 bp	0	59.5 ± 4.2	NA	NA	NA
DNA	11 bp	10	ND	49.4 ± 4.9	ND	1.21
DNA	11 bp	50	4.80 ± 0.23	3.60 ± 0.15	12.4	16.5
DNA	11 bp	100	3.30 ± 0.13	2.50 ± 0.22	18.0	23.8
RNA	5′ss-11 bp	2^h^	20.4 ± 0.7	2.58 ± 0.07	1.56	10.9
mixed	11 bp-2R-9D	0	58.3 ± 3.4	NA	NA	NA
mixed	11 bp-2R-9D	2	28.2 ± 1.4	20.0 ± 0.9	2.07	2.92
mixed	11 bp-3R-8D	0	73.0 ± 2.8	NA	NA	NA
mixed	11 bp-3R-8D	2	25.0 ± 0.9	4.60 ± 0.15	2.92	15.9
mixed	11 bp-4R-7D	0	67.0 ± 5.0	NA	NA	NA
mixed	11 bp-4R-7D	2	24.3 ± 1.2	1.00 ± 0.04	2.76	67.0
mixed	11 bp-8D-3R	0	21.8 ± 1.2	NA	NA	NA
mixed	11 bp-8D-3R	2	15.9 ± 1.4	9.20 ± 0.70	1.37	2.37
mixed	11 bp-7D-4R	0	20.6 ± 1.3	NA	NA	NA
mixed	11bp-7D-4R	2	16.2 ± 0.4	6.00 ± 0.40	1.27	3.43
mixed	11 bp-4D-2R-5D	2	14.8 ± 0.4	16.9 ± 0.7	ND	ND
mixed	11 bp-4D-4R-3D	0	20.1 ± 1.4	NA	NA	NA
mixed	11 bp-4D-4R-3D	2	10.0 ± 0.2	4.70 ± 0.19	2.01	4.28

^a^Oligonucleotides consisted of ribose or deoxyribose residues that formed RNA–DNA or DNA–DNA duplexes on the ssDNA attached to the magnetic beads. The mixed oligonucleotides were deoxyribose residues with two to four ribose substitutions at different positions within the oligonucleotide (see text).

^b^The single-stranded flanking sequence (5′ or 3′) was (A)_11_;

^c^|*T*_off_| is the mean displacement time of the oligonucleotide from the DNA; the values of |*T*_off_| are shown with the standard errors of the regression; values typically represent 100–150 cyclic measurements with about 30–50 beads;

^d^ATP concentration was 1 mM.

^e^The factor of Ded1-dependent enhancement of *T*_off_ over that of the intrinsic *T*_off_ (no protein).

^f^NA: not applicable.

^g^ND: not determined.

^h^A different preparation of Ded1 was used that had slightly higher helicase activity.

We then added 10 nM Ded1 and 1 mM ATP to the reactions (Figure 2B; Table 1). As expected, the blunt-end duplex (11 bp) was the worst substrate for Ded1, which increased the off rate by 3.9-fold (16.9 s/4.30 s) over the intrinsic, protein-independent rate. Unexpectedly, Ded1 increased the off rate of the more stable 5′ss-11 bp oligonucleotide by nearly 22-fold (28.0 s/1.30 s) while the less stable 11 bp-3′ss was enhanced by only 5.9-fold (14.7 s/2.50 s). This contradicted previously published results that duplexes with either 5′ or 3′ extensions were equally effective as substrates. These values were equivalent with Ded1 reducing the duplex energy by one or two base pairs. Increasing the Ded1 concentrations increased the off rates, which was consistent with a rate-limiting step being diffusion-controlled binding of the substrate by the helicase (|*T*_off_|(+ATP), Table 1). Interestingly, Ded1 in the absence of ATP likewise showed a concentration-dependent displacement of the duplex, although significantly less efficiently than with ATP (|*T*_off_|(-ATP), Table 1). However, these ATP-independent values were more dependent on the duplex stability than on the presence of single-stranded extensions (data not shown).

As mentioned above, DEAD-box proteins are thought to be highly sensitive to the duplex stability. Thus, we repeated the previous experiment with oligonucleotides that could form 15 bp RNA–DNA duplexes but with the same flanking sequences, when present. As expected, the 15 bp duplexes were 35- to 70-fold more stable than the 11 bp duplexes in the absence of protein (Table 1). We then tested the stability of the duplexes in the presence of 20 nM Ded1 and 1 mM ATP; the higher protein concentration was used to partially compensate for the much larger values of *T*_off_. Once again, the more stable duplex was displaced more efficiently while the blunt-end duplex was displaced less well, although the off rates between duplexes showed less variability than for the 11 bp duplexes (Table 1). However, the |*T*_off_| values in the presence of ATP were very similar to those obtained for the 11 bp duplexes, even though the former duplexes were much more stable. This was further evidence that the rate-limiting step was partially determined by diffusion-controlled binding of the substrate by the helicase. Thus, Ded1 in the presence of ATP enhanced the intrinsic displacement of the 15 bp duplexes by 250- to 380-fold, which was 15- to 65-fold more than for the same reaction with 11 bp duplexes. This ratio was compatible with the enzyme reducing the duplex size by 2–4 bp (Table 1). In contrast, Ded1 in the absence of ATP had negligible effects on the off rates of the 5′ss-15 bp, which was not the case for the less stable 11 bp duplexes (Table 1).

### DNA duplexes were weak substrates for Ded1

DEAD-box proteins are generally considered to be ATP-dependent RNA binding proteins and RNA-dependent ATPases. Indeed, about a third of the interactions between Vasa and the RNA ligand involve interactions with the 2′ hydroxyl of the ribose sugar (27). However, the remaining two-thirds of the interactions are with the phosphodiester backbone, which are probably stronger. Moreover, some laboratories have reported that DNA duplexes are substrates for other DEAD-box helicases (reviewed by 5). Although dsDNA forms a B-form helix, as opposed to the A-form helix for dsRNA and for RNA–DNA duplexes, it is flexible enough to assume the A form as well. Therefore, we tested the 11 bp oligonucleotide as a DNA–DNA duplex. Typically, RNA–RNA duplexes are more stable than the equivalent DNA–DNA duplexes, but RNA–DNA duplexes are highly variable and strongly dependent on the sequence (28). Consistent with this, the DNA–DNA 11 bp duplex was 3.5-fold more stable (59.5 sec) than the equivalent RNA–DNA duplex (16.9 sec) in the absence of protein (Table 1).

We next tested the DNA–DNA duplex with variable concentrations of Ded1 in the presence or absence of ATP. Ded1 showed only a ∼20% increase (59.5 sec/49.4 sec) in displacement over the intrinsic |*T*_off_| in the presence of ATP and at the 10 nM concentration used with the RNA–DNA duplex (Table 1). We needed to use 5- to 10-fold higher Ded1 concentrations to get comparable values as those obtained with the 11 bp RNA–DNA duplex. However, these values were only slightly faster than the values we obtained in the absence ATP (|T_off_|(+ATP), compared to |T_off_|(-ATP), Table 1). In contrast, the 15 bp RNA–DNA duplex, which was nearly 14-fold more stable (820 s) than the 11 bp DNA–DNA (59.5 s), showed nearly 250-fold (820 s/3.30 s) higher activity in the presence of ATP than in its absence (Table 1 and data not shown). We had previously shown that Ded1 has weak, ATP-independent, affinity for ssDNA (23). This suggested that Ded1 was able to disrupt duplexes largely through a mass action effect that presumably stabilized or promoted the ssDNA. This was consistent with what other investigators found with single-stranded DNA binding proteins; e.g. T4 bacteriophage Gene 32 (29). Thus, it appeared Ded1 was using different binding mechanisms to displace the different duplexes that were either dependent or independent of bound ATP.

### Mixed DNA-RNA chimeric oligonucleotides were variable substrates for Ded1

The previous experiment showed that the ATP-dependent displacement activity of Ded1 was highly dependent on the presence of RNA in at least one of the strands. Previous investigators found that as few as two RNA nucleotides in an otherwise DNA duplex were sufficient for it to function as a substrate for Ded1 (7). We tested this in our assay with 11 bp DNA oligonucleotides that had two to four RNA nucleotide substitutions at various positions. For example, 3R-8D had three ribose nucleotides at the 5′ end of the oligonucleotide and 8D-3R had three ribose nucleotides at the 3′ end. As expected, the intrinsic |T_off_| in the absence of Ded1 was dependent on the position of the RNA nucleotides and subsequent nearest-neighbor energies. We found that duplexes with RNA at the 5′ end of the oligonucleotide were more stable than those at the 3′ (mixed, Table 1).

We next determined the off rates in the presence of Ded1 with and without ATP. In these experiments, a different preparation of Ded1 was used that was found to be slightly more active. Consequently, we recalibrated the protein concentrations used in the assay to give approximately the same |*T*_off_| with the 5′ss-11 bp duplex for the new preparation relative to the previous preparation (2.58 s versus 1.30 s, respectively; Table 1). Regardless, all direct comparisons were made with the same preparation of Ded1. As noted above for the DNA duplex, Ded1 enhanced the off rates of the oligonucleotides in the absence of ATP (|*T*_off_|(-ATP), Table 1). Although the |*T*_off_| values varied, the relative efficiencies were very similar regardless of the position of the ribose nucleotides. In contrast, in the presence of ATP the oligonucleotides with three or four ribose nucleotides at the 5′ end (3R-8D, 4R-7D) were much more efficiently displaced than those with the 3′ ribose (8D-3R, 7D-4R) even though the former formed more stable duplexes (|*T*_off_|(+ATP), Table 1). Ribonucleotides in the middle of the oligonucleotides (4D-2R-5D, 4D-4R-3D) likewise showed inefficient ATP-dependent displacement (Table 1). It may be that ribonucleotides at the 5′ positions promoted an A-form helix that was a better substrate for Ded1, perhaps through binding of RecA-like domain 2.

### Ded1 has high affinity for ssRNA and low affinity for dsRNA

**Figure 3.**
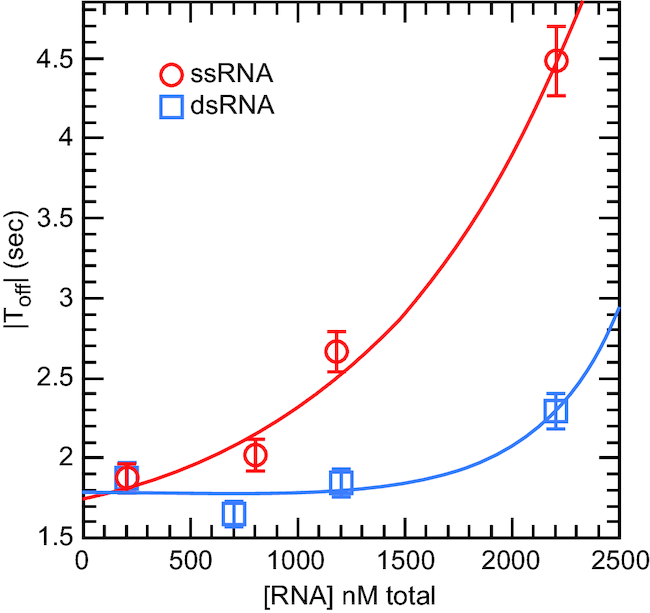
Extraneous RNA inhibited the displacement reaction of Ded1 by sequestering the protein in a nonproductive state. Single-stranded (ssRNA) or double-stranded (dsRNA) RNA was added in increasing amounts to reactions containing 10 nM Ded1, 1 mM ATP and 200 nM of 5′ss-11 bp RNA, which was complementary to the DNA. The 5′ss-11 bp RNA was used at large excess over the fixed DNA, and consequently it also represented an ssRNA competitor for binding Ded1. The ssRNA (RNA01) was a 25-nucleotide-long oligonucleotide with little or no complementarity to the DNA. The dsRNA was made by hybridizing RNA01 to its complementary strand (RNA02). The F_hold_ was at 7 pN. Data were fit to an exponential equation, P(RNA) = *a* + b*exp(*K*_inhibit_*[RNA]), where a and b are constants and *K*_inhibit_ is the affinity for the competitors. The values of |*T*_off_| are shown with the standard errors of the regression.

We have previously shown that Ded1 has low affinity for dsRNA in electrophoretic mobility shift assays (EMSA) and high AMP-PNP-dependent affinity for single-stranded RNA (23). Thus, we expected an added ssRNA, which could not hybridize on the ssDNA, would be an affective competitor for Ded1 binding in the presence of ATP and that this would reduce the observed |*T*_off_| values. We used increasing amounts of a 25-nucleotide-long oligonucleotide (ssRNA01). However, the 5′ss-11 bp RNA (22mer) oligonucleotide that formed the duplex was used in large excess (200 nM) over the fixed ssDNA to ensure complete hybridization, and consequently it also could function as a competitor. As anticipated, Figure 3 shows that ssRNA01 was an effective competitor in the presence of ATP for Ded1. In contrast, a dsRNA made by hybridizing ssRNA01 with ssRNA02, which formed a 25 bp duplex, was a relatively poor competitor. The much higher concentrations needed to see inhibition of Ded1 with dsRNA reflected either low association affinities, high off rates or both.

### ATP hydrolysis was needed to see duplex displacement

**Figure 4.**
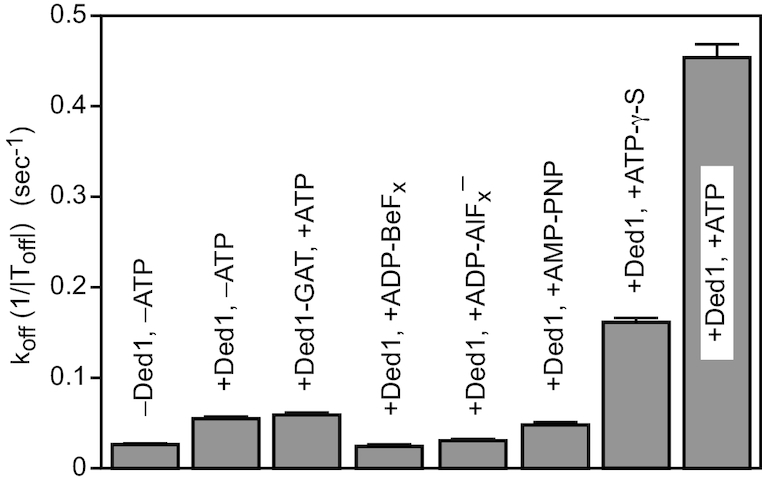
The dissociation rates (1/|T_off_|) of the 5′ss-11 bp RNA oligonucleotide in the presence of Ded1 and different ATP analogs. The intrinsic stability (–Ded1, –ATP) of the duplex is as shown. Ded1-GAT contained a mutation in the Walker A motif (motif I) that inactivated the ATPase activity and eliminated its ATP-dependent affinity for RNA (20). ADP-BeF_*x*_ was made by mixing 1 mM ADP with 3 mM BeCl_2_ and 15 mM NaF. Similarly, ADP-AlF_4_^−^ was made by mixing 1 mM ADP with 3 mM AlCl_3_ and 15 mM NaF. Ded1 was used at 10 nM, and ATP and the analogs were used at 1 mM concentrations when present. The *F*_hold_ was at 6 pN. The mean *k*_off_ is shown with the standard errors of the regression. The lower error bars were deleted for clarity.

It previously was shown that ADP-BeF_*x*_, a nonhydrolyzable analog of ATP, supported the unwinding activity of Ded1 (30). Others have shown that Ded1 requires less than one ATP per unwinding event (31). This implied that ATP hydrolysis was only needed to recycle the protein by converting it into an ‘open’ state with low affinity for the RNA. The release of the ligands (RNA, ADP, and PO_4_^3−^) would prepare Ded1 for a new round of reactions. We tested this with our single-molecule assay using the 5′ss-11 bp oligonucleotide (Figure 4). As previously noted, Ded1 enhanced the intrinsic |T_off_| by 1.6-fold (28.0 s/18.0 s) in the absence of ATP and by 22-fold (28.0 s/1.30 s) in its presence with 10 nM protein (Table 1, Figure 4). We next tested various ATP analogs. Beryllium can take multiple valences, but the forms of BeF_*x*_ are isomorphous with the phosphate group (32). Thus, ADP-BeF_*x*_ is considered an analog of the ground state of ATP. In contrast, MgADP-AlF_4_^−^ is an analog of the transition state of ATP during S_N_2 hydrolysis (32). However, when tested in our assay the compounds actually stabilized the duplexes in the presence of Ded1 (Figure 4). This was not entirely surprising; aluminum cations were expected to stabilize the duplex through the Manning's counterion condensation principle. Although beryllium has high ionization energy and probably does not exist as a free cation, it is known to bind to and aggregate nucleic acids (33). These stabilizing effects probably compensated for the presence of Ded1, which by itself destabilized the duplex slightly.

Ded1 with AMP-PNP enhanced the displacement of the oligonucleotide slightly less well than the protein alone. AMP-PNP is an imperfect analog of ATP because of the different bond angles of phosphorus with nitrogen and those with oxygen. In contrast, adenosine 5-[γ-S]-triphosphate (ATP-γ-S), which replaces a nonbridging oxygen with a sulfur, is often considered a slowly hydrolyzing analog (34). We found that ATP-γ-S stimulated the displacement reaction of Ded1 by about 30% of that obtained with ATP (Figure 4). Ded1 was a recombinant protein expressed in *Escherichia coli*, and there was a risk of contaminating *Escherichia coli* proteins. We controlled for this with a mutation in the Walker A motif (motif I) that changed a lysine into an alanine in the P-loop (Ded1-GAT). This mutant was inactive for the ATPase activity (20). It showed the same slight enhancement of the displacement activity in the presence of ATP as the wildtype Ded1 did in its absence, which showed that the ATP-dependent reactions were indeed caused by Ded1 (Figure 4).

Our results with ADP-BeF_*x*_ were in contradiction with previously published results that indicated that ATP hydrolysis was only needed for recycling the protein. However, the previous experiments with ADP-BeF_*x*_ were done with several orders of magnitude more Ded1 than substrate (30), and those done with ATP involved a weak duplex of 5–6 bp (31). Consequently, these unwinding events were likely inefficient and reflected very low probability events. This implied that multiple Ded1-ATP binding and hydrolysis events were needed for each observed displacement reaction. However, it should be noted that ATP analogs can alter the enzymatic properties of proteins in unanticipated ways (35).

### Ded1 lacked processivity

**Figure 5.**
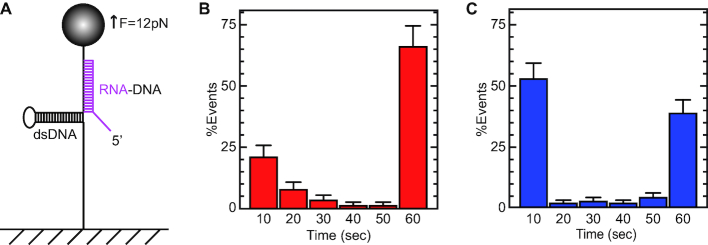
Experimental protocol for determining the processivity of Ded1. (**A**) An RNA oligonucleotide (magenta) was used that could form a 30 bp duplex with the DNA and that had a 5′, 15-nucleotide-long, single-stranded region. The RNA hybridized in the forked region of the DNA hairpin, and consequently the hairpin could only partially reform. An F_hold_ of 12 pN was used to offset the refolding force of the hairpin that would otherwise displace the hybridized RNA (equivalent to step C, Figure 1A). The F_hold_ was maintained for a *T*_hold_ of 60 s. The force was then reduced to zero, and the oligonucleotide was fully displaced for the next round of experiments. (**B**) The distribution of the *T*_off_ values of the oligonucleotide in the absence of protein. Each of the histogram bars represents the mean and standard deviations of oligonucleotides displaced from the DNA at the indicated times that was normalized for the total observed events. The distribution was not uniform; this indicated that the long-lived species were stable during the hold time of 60 seconds and represented fully hybridized duplexes. The shorter-lived species (10–30 s) represented partially hybridized oligonucleotides that were eventually displaced by the refolding DNA hairpin. (**C**) The displacement profile of the hybridized oligonucleotide in the presence of 100 nM Ded1 and 1 mM ATP. Ded1 facilitated the displacement of the partially hybridized oligonucleotides, which resulted in a single large peak at 10 s. In contrast, Ded1 was not able to displace the fully hybridized oligonucleotide during the duration of the *T*_hold_. The mean number of events for each time interval normalized for the total number of observed events is shown with the standard deviations. The lower error bars were deleted for clarity.

We tested for Ded1 processivity with an RNA oligonucleotide that could form a 30 bp duplex with a 5′, 15-nucleotide-long, ssRNA tail (5′ss-30 bp; Figure 5A). In this case, we hybridized the oligonucleotide in the stem region of the hairpin because the |*T*_off_| would be far too long in the apical region. During phase II at an *F*_hold_ of 12 pN, the hairpin spontaneously refolded until it reached the hybridized oligonucleotide. However, the refolding hairpin destabilized the hybridized oligonucleotide relative to the case of an unconstrained duplex. Nevertheless, the oligonucleotide remained bound during the *T*_hold_ of 60 s at an *F*_hold_ of 12 pN. At phase III, the force was reduced to zero (*F*_zero_) to remove any remaining bound oligonucleotide.

We obtained a complicated blocking distribution that reflected long-lived, completely hydridized oligonucleotides at 60 s and various short-lived, partially hybridized species at 10–30 s (Figure 5B). Ded1 in the presence of ATP reduced the short-lived species to the 10 s range, but it left the long-lived species unchanged (Figure 5c). In contrast, Ded1 in the solution reduced the number of completely hybridized RNA oligonucleotides at an *F*_open_ of 20 pN, presumably by binding and sequestering portions of the ssRNA. Thus, Ded1 displaced the weaker duplexes, but it was unable to translocate on the RNA and displace the longer duplex. Moreover, Ded1 was not able to form functional oligomers on the duplex to facilitate its displacement.

### Ded1 displaced blunt-end duplexes even in the absence of ssRNA or ssDNA

**Figure 6.**
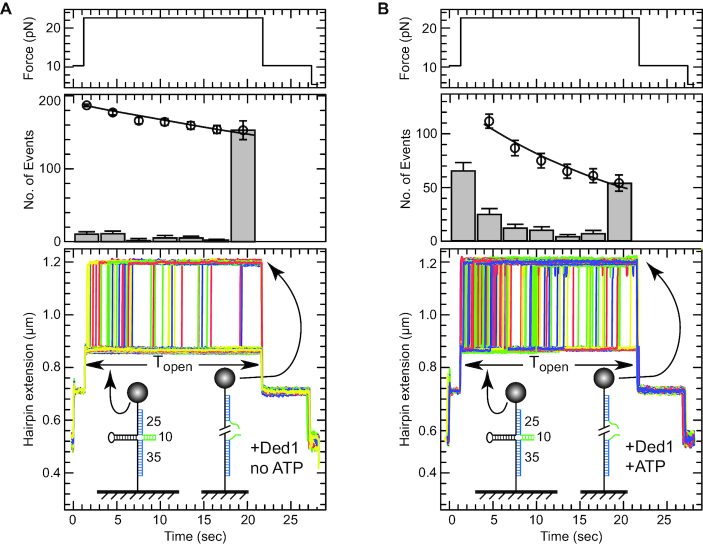
The experimental protocol for determining whether Ded1 recognized blunt-end duplexes. Two mixed RNA–DNA oligonucleotides were used that hybridized at the forked region of the DNA hairpin. The lower oligonucleotide formed a 35 bp duplex and the upper oligonucleotide formed a 25 bp duplex (shown in blue) at *F*_open_ of 20 pN (equivalent to state B, Figure 1A). When the force was reduced to 12 pN (equivalent to state C, Figure 1A), the DNA hairpin refolded until it arrived at the hybridized oligonucleotides. At this point a 10 bp RNA duplex (shown in green) formed between the oligonucleotides. This cruciform was highly stable and resisted hairpin opening for some time (|*T*_open_|) even at 20 pN of applied force. However, the DNA became fully elongated when the RNA duplex was disrupted. (**A**) The top panel shows the applied force, the middle panel the number of events and the bottom panel shows a typical assay in the presence of 2 nM Ded1 alone for six cycles of an attached DNA molecule. The last time point for the number of events represents mostly unreacted molecules. The average time |T_off_| to open the duplex in this case was 79 s with 34 molecule unwound out of 187. (**B**) A typical assay in the presence of 2 nM Ded1 and 1 mM ATP for six cycles of an attached DNA molecule. In the presence of ATP, the Ded1 was able to disrupt the RNA duplex more effectively, thus reducing the mean opening time to 20.6 s with 123 molecule unwound out of 177.

We called the duplexes blunt end when they lacked single-stranded RNA extensions. However, this was not strictly true because of the flanking ssDNA of the extended hairpin template. Moreover, others have shown that at very high Ded1 to substrate concentrations that ssDNA enhanced the enzymatic activity by acting as loading sites (7). We designed another experimental assay to test real blunt-end dsRNA. We hybridized two DNA–RNA mixed oligonucleotides at positions corresponding to the fork section of the extended DNA hairpin. The DNA residues of the oligonucleotides were complementary to the extended DNA and formed 25 and 35 bp duplexes. The RNA residues between the two oligonucleotides were complementary to each other but not to the extended DNA, and they formed a 10 bp RNA–RNA duplex. The result was a cruciform structure with three dsDNA arms, one dsRNA arm and an intermediate extension at an *F*_hold_, of 12 pN (Figure 6).

The cruciform resisted the reopening of the hairpin at 20 pN (state B). The average time, |*T*_open_|, to open the hairpin in the absence of ATP and Ded1 was found to be 96.5 s at 20 pN. Ded1 in the absence of ATP reduced the |*T*_open_| to 79 s, which represented a relative enhancement of 1.2-fold. In the presence of ATP, Ded1 reduced the |*T*_open_| to 20.6 s, which was similar to relative efficiency as the previously used blunt-end 11 bp duplex (∼4.7-fold; Table 1). We concluded that Ded1 could directly bind and disrupt blunt-end RNA duplexes, although less efficiently than those with ssRNA extensions.

## DISCUSSION

We present here a new, highly sensitive method for detecting the displacement of short duplexes by nonprocessive helicases. This assay is based on the large change in extension of a DNA hairpin that is manipulated at the single-molecule level with magnetic tweezers. The method involves hybridizing oligonucleotides in regions corresponding to the apex or forked regions of an extended DNA hairpin under force. When the force is reduced, the hybridized oligonucleotides prevent the hairpin from refolding, which can be easily quantified by differences in the extension length. The time it takes to displace the oligonucleotide and reform the full-length DNA hairpin is given as the mean off time (|T_off_|), which can be measured in the presence and absence of helicases, and under different conditions. The assay is extremely flexible and sensitive, and it can be easily modified so that different parameters can be varied. Moreover, we can analyze dozens of individual molecules simultaneously, which gives us a statistically significant sampling of the reaction events. We validated this experimental method by analyzing the yeast DEAD-box protein Ded1.

To our surprise, we find that the ATP-dependent activity of Ded1 is remarkably insensitive to the absolute stability of the duplexes within a relatively narrow window of duplex lengths. Ded1 in the presence of ATP displaced duplexes that are 35- to 70-fold more stable at essentially the same |T_off_| rates as the weaker duplexes, which is indicative of a reaction that is partially diffusion-limited. Our measurements support a model where Ded1 effectively destabilizes the double helix by the equivalent of two to three base pairs, but it does not translocate. We think that our assay directly shows the mode of action of Ded1: destabilizing short duplexes. We measure up to several hundred-fold enhanced off rates. Our assay with mixed RNA–DNA nucleotides also reveals that Ded1 activity is mostly dependent on at least three to four ribose nucleotides on the 5′ end of the substrate. Short duplexes with 5′ ssRNA flanking regions were the preferred substrates for Ded1. This is consistent with the solved crystal structures of DEAD-box proteins with ligands that show ‘kinking’ of the 3′ end of the RNA with binding (6). In this case, Ded1 binding the 5′ ssRNA region of the bound oligonucleotide places the kink near the duplex region, which would not be the case for a 3′ ssRNA overhang. Similar observations were recently obtained with the human ortholog of Ded1, DDX3 (24). Moreover, the terminal G-A base pair might create a partially ‘kinked’ substrate that had higher affinity for the protein. However, this effect disappeared with longer duplexes, and it appears to be only one of many different pathways that Ded1 uses to unwind duplexes. Although less efficient, we find that Ded1 is also able to directly bind to and disrupt the duplex. Moreover, Ded1 can displace DNA duplexes, although these latter reactions were relatively weak and largely independent of added ATP. In these cases, Ded1 may be functioning as a molecular detergent to destabilize the duplexes (36,37).

The ATP-dependent reactions are much more efficient than those catalyzed in its absence. This is consistent with the much higher binding affinity of Ded1 for ssRNA in its presence and its weak, ATP-independent affinity for dsRNA and ssDNA (23). However, the absence of increased duplex displacement activity in the presence of nonhydrolyzable ATP analogs indicates that a simple binding model is inadequate. It may be that the analogs are imperfect mimics of ATP or that conformational changes are needed during hydrolysis and phosphate release. On the other hand, it has been shown that Ded1 can displace duplexes in the absence of hydrolysis under conditions of very high protein concentrations or of partially stable duplexes (30,31). Thus it appears that the displacement reaction of Ded1 in the presence of ATP might be inefficient, and that multiple binding and hydrolysis events (turnover) might be needed to see unwinding events. In these cases, the reaction rate would be controlled by the diffusion rate of Ded1 binding to the RNA in the presence of ATP and its subsequent release after hydrolysis, which is consistent with the concentration-dependent reactivity of Ded1 seen by us and by others (38).

The multitude of pathways that Ded1 seems to use to displace duplexes is in agreement with the various published experimental results and different experimental conditions. This is consistent with a highly branched and multiple species pathway, where each intermediate is represented by their own micro rate constants (8). The actual pathway would depend on the experimental conditions and substrates, and it would predominately follow the route of least resistance, which would vary with the conditions. Thus, DNA–DNA displacement might be caused by a mass action phenomenon where the protein stabilizes ssDNA at the expense of the dsDNA, which is largely independent of ATP. However, dsRNA and RNA–DNA duplexes are much more efficiently displaced in the presence of ATP, which points to a different reaction pathway. In all the solved crystal structures of DEAD-box proteins, the RNA substrate is kinked by RecA-like domain 1 in a conformation that is not conducive to a duplex in the presence of ATP analogs (6). In principle, this means ATP-bound Ded1 could not directly bind both RecA-like domains to the duplex. However, RNA and DNA duplexes are highly dynamic in solution, and they undergo unstacking and base pair flaying/opening in the μs to ms time scale, depending on the conditions (39). In particular, base pair flaying could occur at the ends of the duplex and would be highly sensitive to the stabilities of these base pairs.

**Figure 7.**
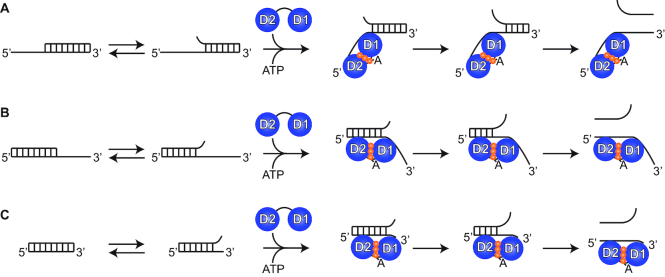
A cartoon of the reaction mechanism. (**A**) The duplex has a 5′ ssRNA overhang, and the duplex shows base pair fraying due to thermal fluctuations. RecA-like domain 2 bind the ssRNA in an A form helix but RecA-like domain 1 kinks the RNA with binding. By so doing it prevents the reformation of the base pairs and additional fraying can occur that ultimately results in duplex displacement. (**B**) The duplex has a 3′ ssRNA overhang. RecA-like domain 2 binds the dsRNA (but only one strand) while RecA-like domain 1 stabilizes the frayed end in a kinked form. This orientation might be expected to be slightly less probable because the binding would have to be right at the junction, since binding further 3′ on the ssRNA would not have any affects. Eventually further fraying results in displacement. (**C**) The duplex has blunt ends. RecA-like domain 2 binds dsRNA (but only one strand) without a problem, but the region that RecA-like domain 1 can bind in the kinked form is rather limited because of the smaller binding site, and hence it is less probable. In all three cases, the binding of RecA-like domain 2 can directly enhance further base pair fraying, and it is possible that direct binding is associated with base pair disruption at the site of binding through a molecular detergent effect (36,37). Ded1 dissociates from the RNA for another round of reactions with ATP hydrolysis and release of the phosphate. In this model, binding can often occur in a nonproductive manner. Hence, ATP hydrolysis and ligand release is important in order for Ded1 to find the correct (productive) orientation. This model involves conserved features found in all DEAD-box proteins and probably represents a common unwinding mechanism.

On the basis of these observations, we propose a model for the unwinding mechanism of DEAD-box helicases as shown in Figure 7. The ATP-bound form of Ded1 recognizes and binds to frayed regions of the duplex and stabilizes them by kinking the RNA bound to RecA-like domain 1 and thereby preventing reformation of the base pairs. Depending on the overall stability of the duplex and the time the protein remains bound, the helix would either further unravel or the protein would fall off and the base pairs would reform. A similar phenomenon occurs with catalytic antibodies (40). The antibodies stabilize reaction intermediates and thereby lower the activation energy barrier. Many enzymes exploit the same strategy (41). By doing so, they can enhance reactions by many orders of magnitude. The ATP-bound form of Ded1 thus stabilizes a reaction intermediate through binding, where the end result depends on the relative energy wells of the substrates and products. Nevertheless, it is clear that no single mechanistic pathway is sufficient to explain all the observed results. Finally, we hope that this new methodology will open new horizons to studying other DEAD-box proteins, as well as other nucleic-acids binding proteins, that have otherwise been difficult to study with single-molecule techniques.

## Supplementary Material

Supplementary Data
