# Supplementary material for: Mechanistic characterization of the DEAD-box RNA helicase Ded1 from yeast as revealed by a novel technique using single-molecule magnetic tweezers
Source: Nucleic Acids Res. 2019 Feb 14;47(7):3699–710. doi: 10.1093/nar/gkz057 (PMC6468243; doi:10.1093/nar/gkz057)
Supplement: Supplementary Data [file gkz057_supplemental_file.pdf]

Supplementary data, Nucleic Acids Research

Mechanistic characterization of the DEAD-box RNA helicase Ded1 from yeast as revealed by a novel technique using single-molecule magnetic tweezers

Saurabh Raj[1,2], Debjani Bagchi[1,2], Josette Banroques[3,4], N. Kyle Tanner[3,4]*, and Vincent Croquette[1,2]*

[1]Laboratoire de physique statistique, Département de physique de l'ENS, École normale supérieure, PSL Research University, Université Paris Diderot, Sorbonne Paris Cité, Sorbonne Universités, UPMC Univ. Paris 06, CNRS, 75005 Paris, France
[2]IBENS, Département de biologie, École normale supérieure, CNRS, INSERM, PSL Research University, 75005 Paris, France
[3]Laboratoire d'Expression Génétique Microbienne, CNRS UMR8261/Université Paris 7-Diderot, Sorbonne Paris Cité Universités, 13 rue Pierre et Marie Curie, Paris, France.
[4]Institut de Biologie Physico-Chimique, PSL Research University, 75005 Paris, France

*To whom correspondence should be addressed. Tel: +33 1 44 32 34 92; Fax: +33 1 44 32 34 33; Email: vincent.croquette@lps.ens.fr
Correspondence may also be addressed to kyle.tanner@ibpc.fr

MATERIALS AND METHODS

Experimental system.

The design of the hairpin DNA and its attachment to the magnetic bead and glass slide are as shown in Figure S1 and as previously described (1).

At F = 5pN hybridization is insensitive to the force applied to the hairpin.

When an oligonucleotide hybridizes with the hairpin in the elongated situation, the hybridization process does depend on the force applied on the hairpin (Fig 2S). At low force (F = 2 pN), the ssDNA is shorter than the dsDNA; thus upon hybridization the extension of the hairpin increases. In that situation, the force is helping the hybridization process. If the force applied to the hairpin is high (F = 10 pN), then the ssDNA is longer than the dsDNA; upon hybridization the hairpin extension shrinks meaning that the hybridization process is hindered by the applied force. If we set the force at 5 pN, the extension of ssDNA and dsDNA is the same; thus the hybridization of the oligonucleotide is neither favored nor hindered by the applied force. Around the point F = 5 pN, we can evaluate the energy opposed or delivered by increasing or decreasing the force by 1 pN; it correspond to ~0.2 pN·nm that is 1.6% of the typical base pair energy.

Various schemes used for measuring the kinetics of Ded1 in our experiments.

Below we sum-up the various schemes that were used in measuring the kinetics of Ded1 helicase in our system. RNA nucleotides are written as A, U, G and C, while DNA nucleotides have a "d" prior to each base to distinguish them from RNA bases. The complimentary regions of the oligonucleotides are underlined.

Scheme 1: Hybridization of oligonucleotides to the loop of DNA hairpin. The schematic is shown in Figure 1. The force at which the binding time (T_{off}) of the RNA oligonucleotide was calculated is such that both dsDNA and ssDNA extensions are similar. As such, the affect of external force is minimal on the DNA-RNA hybrid. This scheme was used in various cases as elaborated below:

a) Determination of loading strand preference. We used 11 bp-long RNA oligonucleotides, with and without noncomplementary overhangs, to bind to the loop of DNA hairpin described above.

11 bp: 5' AGA UGC CAA AA 3'

5'ss-11 bp: 5' AAA AAA AAA AAA GAU GCC AAA A 3'

11 bp-3'ss: 5' AGA UGC CAA AAA AAA AAA AAA A 3'

b) Effects of the polarity on longer RNA-DNA duplex. 15 bp-long RNA oligonucleotides were used in the configuration as described above to determine: a) whether Ded1 was able to melt much stronger duplexes, and b) how does the polarity affect longer duplexes.

15 bp: 5' C GUC AGA UGC CAA AA 3'

5'ss-15 bp: 5' AAA AAAAAA AAC GUC AGA UGC CAA AA 3'

15 bp-3'ss: 5' CGU CAG AUG CCA AAA AAA AAA AAA AA 3'

c) Effects of Ded1 on DNA-DNA duplex. To test whether Ded1 has any affect on dsDNA, we used an 11 bp-long DNA oligonucleotide that bound to the loop of the DNA hairpin. The DNA oligonucleotide had a sequence of:

DNA-11bp: 5' dAdGdA dTdGdC dCdAdA dAdA 3'

d) Effects of Ded1 on chimeric RNA-DNA oligonucleotide. To determine whether Ded1 works on chimeric RNA-DNA oligonucleotides and to determine the minimum number of ribose nucleotides required, we designed oligonucleotides with a mixture of RNA and DNA bases. Following are the oligonucleotides used:

11bp-2R-9D: 5' AGdA dTdGdC dCdAdA dAdA 3'

11bp-3R-8D: 5' AGA dTdGdC dCdAdA dAdA 3'

11bp-4R-7D: 5' AGA UdGdC dCdAdA dAdA 3'

11bp-8D-3R: 5' dAdGdA dTdGdC dCdAA AA 3'

11 bp-7D-4R: 5' dAdGdA dTdGdC dCAA AA 3'

11 bp-4D-2R-5D: 5' dAdGdA dTGC dCdAdA dAdA 3'

11bp-4D-4R-3D: 5' dAdGdA dTGC CAdA dAdA 3'

Scheme 2: Hybridization of RNA oligonucleotide to the stem of the DNA hairpin substrate. To determine whether Ded1 possessed any processivity, we used a slightly longer RNA oligonucleotide with an overhang at the 5' end. This oligonucleotide was designed to bind to the stem of the hairpin, rather than the loop, because the T_{off} at the loop would have been too high to be of any practical use in the current experiments. At the stem, the hairpin-closing fork pushed at the oligonucleotide and hence destabilized the bound oligonucleotide. The scheme used is detailed in Figure 5. The oligonucleotide used in this case was:

5'ss-30 bp: 5' AAA AAA AAA AAA AAA GAU AAG CCU ACU ACA GUA GAU UUU GAC GGG 3'

Scheme 3: Hybridization of DNA-RNA chimeric oligonucleotides to the two strands of the DNA hairpin to form a cruciform structure. There was no true blunt end in the true sense in our experiments with oligonucleotides bound at the apical region of the hairpin. The flanking DNA strands on either side of the hybridized oligonucleotide could also provide a potential loading site for unwinding the blunt-end RNA oligonucleotide (e.g., for 11 bp and 15 bp in scheme 1a and 1b). To remove any doubts, we designed chimeric oligonucleotides that formed a cruciform structure (Figure 6). This cruciform created a 10 bp RNA duplex without any single-stranded region (RNA or DNA) as a loading site. The following oligonucleotides were used for this purpose:

Cruciform1: 5' GAG CGU CAG CdCdC dAdCdC dAdTdT dCdAdC dAdTdG dCdTdT dAdGdG dAdGdC dGdG 3'

Cruciform2: 5' dCdGdG dTdTdA dGdTdT dTdCdC dGdCdT dCdCdT dAdAdG dCdAdT dGdTdG dAdAdT dGdGdT dGdGG CUG ACG CUC 3'

REFERENCES

1. Manosas, M., Spiering, M.M., Zhuang, Z., Benkovic, S.J. and Croquette, V. (2009) Coupling DNA unwinding activity with primer synthesis in the bacteriophage T4 primosome. *Nat Chem Biol*, **5**, 904-912.

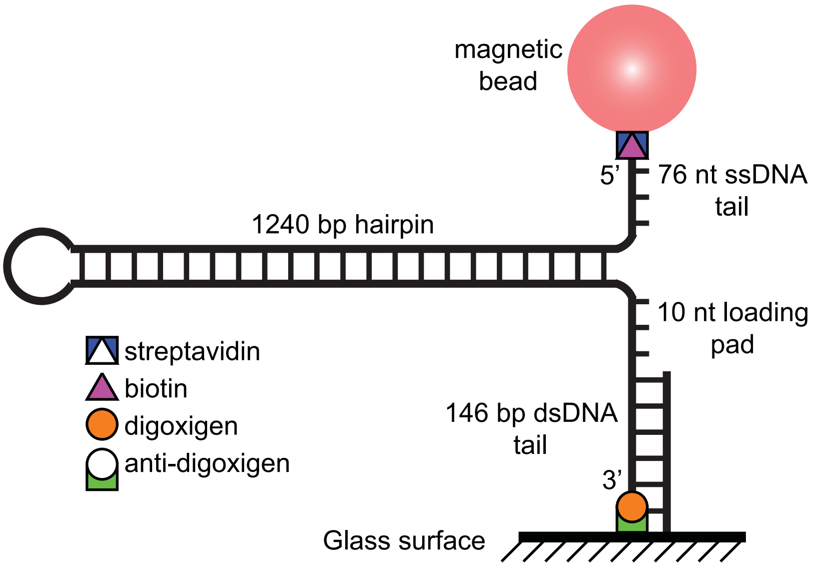

Supplemental Figure S1. DNA hairpin substrate used in most of the experiments of this work. A dsDNA template 1240 bp long was closed by a four nt loop at one end and a fork at the other. The 5' end of the fork ended with a 76 nt ssDNA tail that was not complimentary to the rest of the molecule, and it had a biotin at the extremity. This biotin bound to the streptavidin-coated magnetic beads. The 3' end of the fork encompassed a short 10 nt ssDNA loading pad followed by a 146 dsDNA tail with six 3' digoxygenins, which bound to the anti-digoxygenin-coated micro-channel on the cover slip. This 3' end was created by partially hybridizing a 146 nt-long oligonucleotide at the 3' end of the hairpin DNA, and then the hairpin DNA was elongated with T4 DNA polymerase in the presence of dATP, dCTP and dUTP-digoxigenin using the unhybridized portion of the 146 nt-long oligonucleotide as a template as previously described (1).

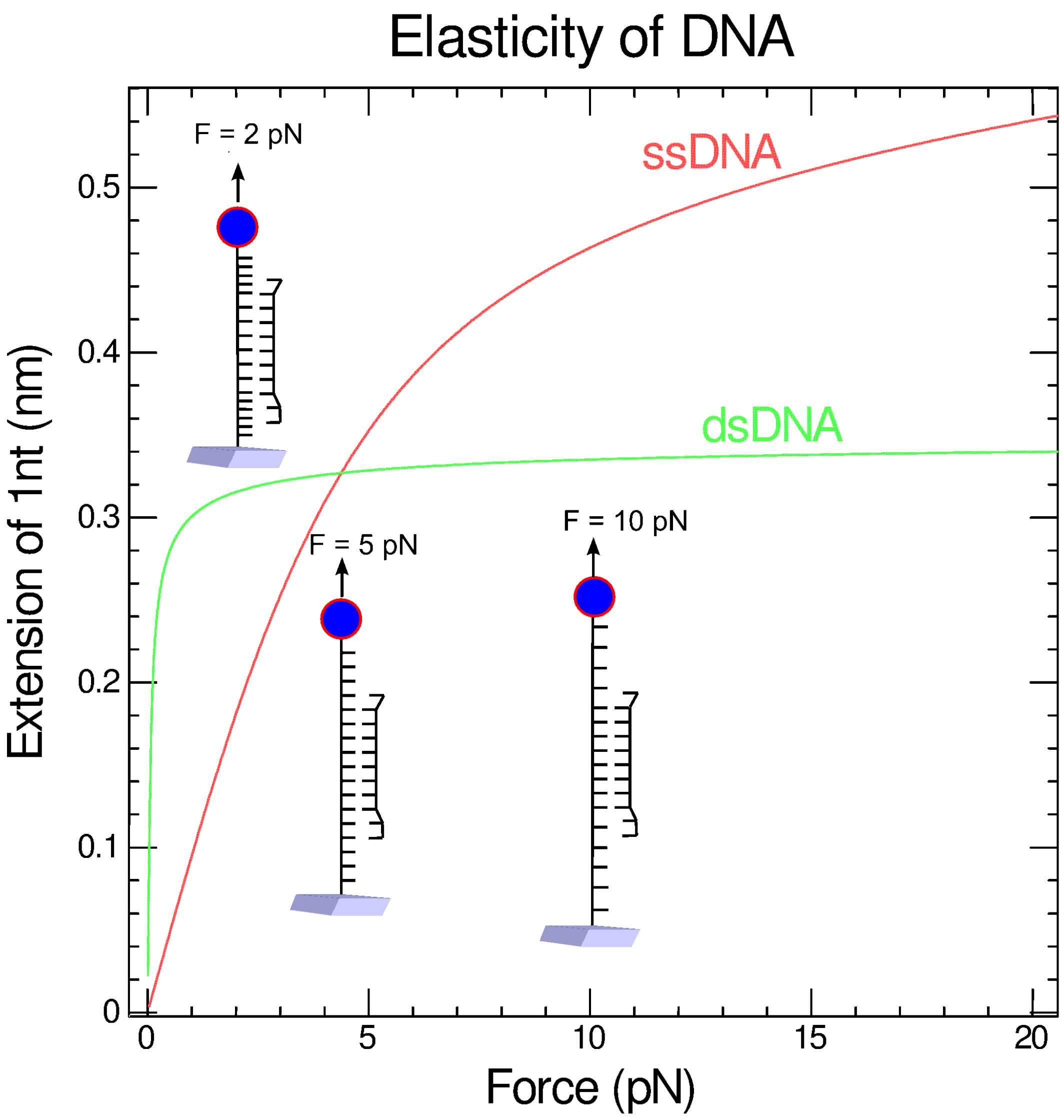

Supplemental Figure S2. Elastic behavior of ssDNA and dsDNA and its consequence on the oligonucleotide hybridization.

The green curve displays the extension of a single base pair versus the applied force F. This curve is described by the Worm-Like Chain model with a persistence length of 50 nm. The dsDNA extension reaches its maximum length with a very weak force in the range of one or two pN. On the other hand, ssDNA is not described by a simple polymer model, but it presents a far smaller persistence length. As a result, the extension of ssDNA evolves on a larger range of force. Furthermore, ssDNA does not need to wrap around another strand as dsDNA does and thus can extend much more that the dsDNA. The two elasticity curves meet around 5 pN, with ssDNA being shorter than dsDNA below 5pN and longer above.
